# Incidence and predictors of organ failure among COVID-19 hospitalized adult patients in Eastern Ethiopia. Hospital-based retrospective cohort study

**DOI:** 10.1186/s12879-022-07402-6

**Published:** 2022-04-28

**Authors:** Abdi Birhanu, Galana Mamo Ayana, Bedasa Taye Merga, Addisu Alemu, Belay Negash, Ahmed Seid, Yadeta Dessie

**Affiliations:** 1 School of Medicine, College of Health and Medical Sciences, Haramaya University, Harar, Ethiopia; 2 School of Public Health, College of Health and Medical Sciences, Haramaya University, Harar, Ethiopia; 3 Hiwot Fana Specialized University Hospital, College of Health and Medical Sciences, Haramaya University, Harar, Ethiopia

**Keywords:** COVID-19, SARS-CoV-2, Predictors, Organ failure, Comorbidity, Aged, Smoking, Ethiopia

## Abstract

**Background:**

Organ failure is incapability of at least one of the body organs to carry out a normal body functions. Identifying the predictors of the organ failure is crucial for improving COVID-19 patients’ survival. However, the evidence related to this information is not well-established in developing countries, including Ethiopia. Therefore, this study aimed to determine the incidence and predictors of organ failure among adult patients admitted to Hiwot Fana Specialized University Hospital (HFSUH) COVID-19 treatment center from 1st May 2020 to 20th August 2021, Eastern Ethiopia.

**Methods:**

A hospital-based retrospective cohort study design was implemented. Descriptive measures such as mean with standard deviation (SD), median with interquartile range (IQR), percentages, and frequencies were computed. The binary logistic regression was used to identify the association between outcome variables (organ functional status) and independent variables with an adjusted odds ratio (AOR) at a 95% confidence interval. A significance level was declared at a p-value of less than 0.05.

**Results:**

The mean age of study participants was 47.69 years with the standard deviation (SD) of ± 17.03. The study participants were followed for the median time of 8 days with IQR of 4, 14. The incidence of organ failure was 11.9 per 1000 person-day contribution (95% CI: 9.5, 14.9). Predictors such as age above 60 years (AOR = 1.71, 95% CI: 1.44, 4.53), smoking history (AOR = 5.07, 95% CI: 1.39, 8.15), cardiovascular disease (AOR = 5.00, 95% CI: (1.83, 11.72), and critical clinical stages of COVID-19 (AOR = 5.42, 95%: 1.47, 14. 84) were significantly associated with organ failure among COVID-19 hospitalized patients.

**Conclusions:**

The incidence of organ failure was 11.9 per 1000 person-day contribution. Age, smoking, comorbidity, and clinical stages were significantly associated with organ failure among COVID-19 hospitalized cases. Therefore, clinicians should stringently follow the patients experiencing modifiable predictors of organ failure, especially patients with comorbidities and severe clinical stages. Moreover, the prevention programs that target elders and smokers should be strengthening to save this segment of populations before suffering from organ failure following COVID-19.

**Supplementary Information:**

The online version contains supplementary material available at 10.1186/s12879-022-07402-6.

## Background

The novel coronavirus disease-2019 was recognized in Wuhan China in December 2019 [1]. The causative virus SARS-CoV-2 has been spreading throughout the countries in the globe and was distinguished as a pandemic in March 2020 [2, 3]. A coronavirus commonly spreads from infected people to healthy individuals through infected air, making contact with fingers that contain the virus and then touching their eyes, nose, or mouth with unclean hands [2]. The pandemic, novel coronavirus disease 2019 (COVID-19) led to an unprecedented number of mortalities by causing damage of one or more organs of infected patients [4, 5]

The majority of COVID-19 cases (52%) developed respiratory disorders particularly, the acute respiratory distress syndrome [6]. Another complication such as heart failure, renal failure, liver damage, shock, and other organs failure triggered the early death of COVID-19 patients. The presence of comorbidities and clinical findings such as lymphocytopenia, elevated D-dimer, high fibrin degradation products, and disseminated intravascular coagulation (DIC) could lead to death. In addition, deep vein thrombosis (DVT), venous thromboembolism, pulmonary embolism (PE), systemic and pulmonary arterial thrombosis, embolism, ischemic stroke, and myocardial infarction (MI) are other commonly diagnosed clinical conditions that worsen the poor prognosis of COVID-19 patients [7, 8].

Moreover, another studies revealed that the COVID-19 clinical course could be complicated and end up with organ failure and death by factors such as gender, age (> 60 years), comorbidities such as hypertension, diabetes, cardiovascular disease, acute respiratory distress syndrome (ARDS), could end up with organ dysfunctions and finally dead [9, 10]. Particularly, the risk factors associated with the development of ARDS may easily deteriorate the health of COVID-19-infected patients [11]

In order to prevent the organs failure, providing heated high flow oxygen, non-invasive ventilation, intubation, mechanical ventilation, vasopressors, and dialysis are common clinical managements being implemented in different clinical settings [12]. Therefore, distinguishing the burden of organ injuries due to COVID-19 is required for the betterment of clinical management of the patients. Additionally, understanding the predictors of organ failures is vital to identify high risky patients during pharmacological and non-pharmacological therapies provision. Despite that knowing the contributing factors of organ failure among COVID-19 patients could significantly contribute in the improvement of patient survival status, there is dearth of literatures in the resource-limited setups like Ethiopia. Therefore, this study aimed to assess the organ failure incidence and predictors amongst COVID-19 patients admitted to Hiwot Fana Specialized University Hospital.

## Methods

### Study setting and design

A hospital-based retrospective cohort study design was carried out in Harari Regional state in the Eastern part of Ethiopia. The regional state is situated on about 522 km away from the Capital city of Ethiopia (Addis Ababa) to the east. The region contains two public Hospitals that comprise Jogula General Hospital and *Hiwot Fana* Specialized University Hospital (HFSUH). This study was conducted in the HFSUH. In the Easter part of the country, the hospital serves as the largest teaching and referral center with about a total of 600 beds. The hospital manages the referral cases received from the Eastern part of Oromia Region, Somali region, Harari region, and Dire Dawa City administration. The hospital has been labeled as COVID-19 treatment center by Ethiopian Minster of Health (MoH) since the outbreak of COVID-19 in Ethiopia [13]

### Participants and eligibility criteria

An adult patient admitted to Hiwot Fana Specialized University Hospital with the diagnosis of COVID-19 between May 2020 and August 2021 was included in this study. The patient whose information was incomplete for the variable of interests was excluded from the study (Additional file 1).

### Sample size determination

The sample size was determined using proportion with the following formula:

n = (zα/2)2 (p.q)/d^2^, where z = 95% CI, p = 70% [14] (Proportion of organs failure among COVID-19 patients), q = 1-p, d = 0.05 (margin of error). n = (1.96)2. (0.7)(0.3)/0.05)^2^. = 323. Finally, by considering 10% of the possible proportion of withdrawals, the sample size was determined to be 355.

### Operational definitions and variables in the study

An organ failure (Organ failed (yes/no) among COVID-19 adult patients was a dependent variable of this study. The independent variables included sex, age, educational status, occupational status, marital status, residence, COVID-19 manifestations, clinical outcomes, utilization of mechanical ventilation, clinical management, laboratory findings, and comorbidities like Diabetic Mellitus (DM), hypertension, stroke, cardiovascular disorders, COPD, asthma, cancers, and HIV/AIDS were some of the commonly assessed diseases. Besides, alcohol consumption, medication history, and current smoking history were evaluated [15]

COVID-19 clinical stages are defined as follows;

Asymptomatic infection*;* COVID-19 confirmed patients who test positive for SARS-CoV-2 using a virology test but who have no symptoms that are consistent with COVID-19. Mild illness; COVID-19 confirmed patients who have any of the various signs and symptoms of COVID-19 such as headache, vomiting, fever, sore throat, cough, muscle pain, nausea, diarrhea, malaise, and losing ability to taste and smell but who do not have shortness of breath, dyspnea, or abnormal chest imaging. Moderate illness; COVID-19 confirmed patients who show evidence of lower respiratory disease during clinical assessment or imaging and who have oxygen saturation (SpO2) greater than or equal to 94 percent on room air at sea level. Severe illness; COVID-19 confirmed patients who have SpO2 less than 94 percent on room air at sea level, a ratio of arterial partial pressure of oxygen to fraction of inspired oxygen less than 300 mm Hg, respiratory count above 30 breaths/min, or lung infiltrates greater than 50 percent. Critical illness**;** COVID-19 confirmed patients who have respiratory failure, septic shock, and/or multiple organ dysfunctions. In this study, critical cases were defined as the cases that fulfilled the definition criteria of severe and critical illness, while non-critical patients were who fulfilled the definition criteria of both mild and moderate illness [16]

Organ failure is defined as incapability of at least one of the organ to carry out normal body functions. This failure includes at least one of lung failure, acute liver failure, acute kidney injury, cardiovascular disease, and wide spectrum of hematological abnormalities and neurological disorders as recognized by a physician [17]

### SARS-COV-2 assaying procedures using RT-PCR

Laboratory examination materials such as Viral transport medium (VTM), swab sticks, ice-box, ice-pads, tongue depressor, marker, requisition form, and personal protective equipment (PPE) were prepared and used [18]. VTM used contains 3 ml fluids composed of gelatin and antimicrobial agents in a buffered salt solution. It was used to prevent the sample collected from drying, maintains the viability of the virus, and avoids the growth of contaminants. The swabs are made from rayon with plastic shaft. A Cotton or calcium alginate swab was avoided to be used since it may inhibit PCR reaction. Ice-box and ice-pads are used for maintaining a cold-chain during sample transportation from sample collection area to laboratory center. The ice-pad was filled with water and stored in the freezer (−20°°C) before and after use [19–21]

### Data collection method and produces

Five Bachelor of Science (BSc) holders nursing professionals were involved in the data collection. Also, two public health professionals were involved for supervising the data collection processes. Before the actual data collection, the data collectors took two days of training on the objective and relevance of the study. Regarding the SARS-CoV-2 sample testing, the standard procedure of SARS-COV-2 sampling and testing procedures were strictly followed and used. The sample contamination from the nasal vestibule was avoided by sterile opening of the outer case of the swab, inserting the swab into the mouth by slightly elevating the tip of the nose, and keeping the tip of the swab in the oropharynx for a few seconds, then rotated to achieve the highest absorption of *oropharyngeal* secretions [22, 23]

### Statistical analysis

Data were entered into Epi-data version 3.1 and exported to STATA version 14.2 for analysis. Descriptive measures such as mean with standard deviation, median with interquartile range (IQR), percentages, and frequencies, and incidence density were performed. The binary logistic regression was used to identify the association between outcome variables (organ failed (Yes/No) and independent variables with an adjusted odds ratio (AOR) at a 95% confidence interval. Statistically, a significance level was declared at a p-value of less than 0.05.

### Ethics approval and consent to participate

Research Ethical Review Committee (IHRERC) of Haramaya University College of Health and Medical Sciences ethically cleared this study. The study was conducted in accordance with the Declaration of Helsinki. Permission was obtained from Hiwot Fana Specialized University Hospital management. Due to the nature of retrospective study, it was difficult to reach the study participants to get informed consent directly from the patients. Instead, the written informed consent was obtained from the hospital head on behalf of the study participants to take information from the patients’ medical records anonymously guaranteeing the information confidentiality. All the data extraction procedures were conducted per the declaration of Helsinki.

## Results

### Socio-demographic and behavioral characteristics

A total of 355 hospitalized adult patients in the HFSUH COVID-19 treatment center between 1^st^ May 2020 and August 2021 were included in this study. The mean age of the study participants was 47.69 years with the standard deviation (SD) of ± 17.03. Nearly, three out of five patients (219/355, 61.69%) were males, while the rest were females. The majority of the study participants (303/355, 85.35%) were married. More than half (214/355, 53.6%) of the study subjects were employed. More than one-third (143/355, 40.28%) of the patients had current smoking history. Nearly one-fourth of the study subjects (93/355, 26.20%) patients had an alcohol drinking history (Table 1).

**Table 1:** Socio-demographic and behavioral characteristics of COVID-19 patients admitted to *Hiwot Fana* specialized University Hospital COVID-19 treatment center, *Harar*, Ethiopia

Variables	Frequency (%)
Sex	
Male	219 (61.69)
Female	136 (38.31)
Age group	
1–60	279 (78.59)
> 60	76 (21.41)
Ethnicity	
Harari	206 (58.03)
Oromo	140 (39.44)
Other	9 (2.51)
Marital status	
Single	41 (11.55)
Married	303 (85.35)
Divorced	4 (1.13)
Widowed	7 (1.97)
Occupational status	
Employed	214 (60.28)
Unemployed	141 (39.72)
Smoking status	
Yes	143 (40.28)
No	212 (59.72)
Alcohol drinking status	
Yes	93 (26.20)
No	262 (73.80)

Other: Somali, Amhara

### Presence of comorbidities and clinical findings

The mean respiratory rate of the patients was 31.41 breaths per minute with the Standard deviation (SD) of ± 10.29. The average heart rate of the study participants was 100.52 beats per minute (bpm) with the SD of ± 20.31. The mean Serum creatinine (mmol/L) was 1.32 with SD of ± 1.46. The mean lymphocyte count** (**1000/µL) was 18.05 with an SD of ± 14.27**. **One hundred-seventy three patients had a history of taking chronic medication before being hospitalized with COVID-19. Regarding the COVID-19 symptoms, the majority of the patients presented with cough, fever, and shortness of breathing by 279/355 (78.59%), 258/355 (72.68%), and 243/355 (68.45%), respectively. Majorly the comorbidities were hypertension, Diabetic Mellitus, cardiovascular disease, and renal disease among 83/355(23.38%), 92/355(25.92%), 68/355 (19.15%), and 24/355 (6.76) patients, respectively. Eighty six (24.29%) of the study participants were in the critical COVID-19 clinical stage, while 54 (15.25%) were in severe COVID-19 conditions. During their hospital stay, 64 (18.03%) of the patients received dexamethasone medication. From the overall admitted patients, 96/355 (27.04%) of them died, while the rest 259/355 (72.96%) of them survived (Table 2).

**Table 2:** Clinical characteristics and comorbidities of COVID-19 patients admitted to Hiwot Fana specialized university hospital treatment center, Eastern Ethiopia

Variables	Frequency (%)
COVI-19 clinical stage	
Critical	86 (24.29)
Not critical	268 (75.71)
Medication history	
Yes	173 (48.73)
No	182 (51.27)
Cough	
Yes	279 (78.59)
No	76 (21.41)
Fever	
Yes	258 (72.68)
No	97 (27.32)
Shortness of breath	
Yes	112 (31.55)
No	243 (68.45)
Hypertensive	
Yes	83 (23.38)
No	272 (76.62)
Diabetic	
Yes	92 (25.92)
No	263 (74.08)
Cardio vascular disease	
Yes	68 (19.15)
No	287 (80.85)
Renal disease	
Yes	24 (6.76)
No	331 (93.24)
Receiving dexamethasone	
Yes	64 (18.03)
No	291 (81.97)
Covid-19 outcome	
Discharged	259 (72.96)
Death	96 (27.04)
Partial oxygen saturation(mmHg)	81.10 (SD ± 16.63)
Respiratory rate (breath/minute)	31.41 (SD ± 10.29)
Heart rate (bpm)	100.52 (SD ± 20.31)
Serum creatinine (mmol/L)	1.32 (SD ± 1.46)
Lymphocyte count (1000/µL)	18.05 (SD ± 14.27)

### Incidence and predictors of organ failure

The incidence of organ failure was 11.9 per 1000 person-day contribution (95% CI: 9.5, 14.9). The patients were followed for a minimum of 1 and a maximum of 74 days with a median follow-up time of 8 days [IQR, (4, 14)]. The proportion of patients with organ failure was 52/355, 14.6%. The organ failure included respiratory failure, renal failure, and cardiac failure. In the multivariable logistic regression model, age, smoking status, developing CVD, and COVID-19 critical clinical stage were statistically significant with organ failure (p < 0.05). Patients who were in the age group of above 60 years developed organ failure 1.71 times more likely than younger people (AOR = 1.71, 95% CI: 1.44, 4.53). COVID19 hospitalized patients who had a smoking history had a higher risk of developing organ failure 5.07 times more likely compared to non-smoker individuals (AOR = 5.07, 95% CI: 1.39, 8.15). Patients who presented with CVD had a risk of developing organ failure 5.00 times more likely than patients presented without comorbid cases (AOR = 5.00, 95% CI: (1.83–11.72). Patients who had critical clinical stages of COVID-19 cases had a risk of developing organ failure 5.4 times more likely compared to non-critical stages.(AOR ═5.42, 95%: 1.47, 14. 84) (Table 3).

**Table 3:** Predictors of organ failure among Covid-19 patients in Hiwot Fana specialized university hospital treatment center, Harar, Ethiopia

Variables	Organ failure		COR (95% CI)	AOR with 95% CI
	Yes	No		
Age category				
18–60	34	245	1	1
> 60	18	58	18.55 [9.05, 38.01]	1.71 [1.44, 4.53]*
Smoking status				
Yes	40	103	6.47 [3.25, 12.87]	5.07 [1.39, 8.15]*
No	12	200	1	1
Alcohol utilization				
Yes	24	69	2.90 [1.58, 5.33]	0.89 [0.35, 2.27]
No	28	234	1	1
Renal disease				
Yes	10	14	4.91[2.05, 11.77]	1.56 [0.56, 2.31]
No	42	289	1	1
Hypertensive				
Yes	33	50	8.78 [4.63, 16.68]	1.76 [0.59, 5.29]
No	19	253	1	1
Diabetic				
Yes	20	72	2.53 [1.33, 4.80]	0.47 [0.16, 1.38]
No	26	237	1	1
Cardiovascular disease				
Yes	30	36	14.21 [7.06, 28.61]	5.00 [1.83,11.72]*
No	16	273	1	1
COVID-19 clinical stage				
Critical	36	50	18.57 [8.65, 39.85]	5.42 [1.47, 14.84]*
Noncritical	10	258	1	1
Respiratory rate(bpm)	–	–	1.13 [1.09, 1.17]	1.04 [0.98, 1.10]
Heart rate(bpm)	–	–	1.05 [1.03, 1.07]	1.03 [0.55, 1.06]
Partial Oxygen saturation(mmHg)	–	–	0.94 [0.92, 0.96]	1.02 [0.98, 1.07]
Temperature (°C)	–	–	4.21[2.78, 6.37]	1.38[0.83, 2.28]

*: Statistically significant variable

## Discussions

The study finding is lower than the findings from Shanghai (17%) [17, 24] and England (25%) [14] This discrepancy could be due to sociodemographic characteristics variation of the study population. In addition, the inclusion of an adequate sample in the present study may bring these differences as the former studies were conducted on a smaller sample size.

Patients who were in the age above 60 years developed organ failure 1.7 times more likely than younger people. Age is the most important non-modifiable sociodemographic characteristic that can affect the health status of an individual. As age increases, the kidney gradually drops its functions [25], maximum oxygen consumption declines, systolic pressure at rest increases [26], unfavorable respiratory mechanics related to reduced expiratory flows, high air trapping, and closing volume, and reduced gas interchange could be happened [27]. Studies showed that age has multiple effects on COVID-19 patients by aggravating the poor progression of the clinical outcomes that may result in organ failures and end up with death [28–31] Therefore; these findings confirmed that elder individuals are at risk of developing severe COVID-19 clinical characteristics that can be manifested by organ failures.

COVID19 hospitalized patients who had a smoking history had a higher risk of developing organ failure which was about 5 times more likely compared to non-smoker individuals. Smoking is a risk factor for several diseases and conditions, including heart and lung disease, cancer, and oral diseases such as periodontal [32]. On top of  COVID-19 effects, smoking may exacerbate the severity of disease progression in causing organ failures complications [33, 34]. Moreover, Nicotine increases the vulnerability of the patients to COVID-19 and aggravates the disease progression through up-regulating the ACE2 expression [35]. Thus, the aggravations of clinical stages by smoking may cause organ failures.

Patients who presented with CVD had the risk of developing organ dysfunctions 5 times more likely than patients who presented without comorbid cases. It’s a fact that comorbidities have a strong association with the multi-organ damages among COVID-19 [36]. Particularly, the studies from China [37–41] showed that cardiac cases had a strong association with the COVID-19 clinical progression. This can be due to the organs which have been affected by comorbid cases before the onset of COVID-19 can be easily prone to damage.

Patients who had critical COVID-19 clinical stages had a risk of developing organ failure that was about 5.4 times more likely than those who had non-critical clinical stages. The advancement of COVID-19 clinical stages is probably triggered by the endothelial damage and thromboinflammation associated with the problem of the immune response [42, 43]. As clinical stages, progress to critical, cellular, and tissue level damages provoke the organs changes that induce multiple organ dysfunctions. The primary strength of this study could be the originality of this study for indicating the organ failure burden among COVID-19 hospitalized patients in resource-limited setting. This pioneer information for resource-limited setups might have clinical importance for the clinicians to closely following the patients with the organ failure predictors to prevent early death. The utilization of robust methods might make the study with stronger evidence than previously conducted studies. Moreover, the strength of this study could be its representativeness due to its adequate sample size enrollment compared to previous studies conducted in other countries. Even if the study has paramount strength, it could not be free from the limitation. This limitation could be that the expected organ failure among COVID-19 infected patients after discharge was not measured in this study. In addition, due to the nature of the retrospective study, the inclusion of all important variables could be difficult.

## Conclusions

The incidence of organ failure was 11 0.9 per 1000 person-day contribution. Age, smoking, comorbidity, and clinical stages were significantly associated with organ failure among COVID-19 hospitalized patients. The development of organ failure could be an early warning sign of poor clinical prognosis of COVID-19 patients. Therefore, clinicians should strictly follow the patients experiencing modifiable predictors of organ failure, particularly patients with comorbidities and severe clinical stages. Moreover, the COVID-19 prevention programs targeted to elders and smokers should be strengthening to save these groups of the population before suffering from organ failures following got ill from COVID-19.

## Supplementary Information

**Additional file 1.** Data collection tool.

## Data Availability

The dataset used and/or analyzed during the current study are available from the corresponding author on reasonable request.

## Abbreviations

AOR: Adjusted Odds ratio
ACE2: Angiotensin converting enzyme 2
CVD: Cardiovascular Disease
IQR: Interquartile range
SARS-CoV-2: Severe Acute Respiratory Syndrome Coronavirus-2
COVID-19: Coronavirus Disease 2019
SD: Standard deviation
