# Supplementary material for: Incidence and predictors of organ failure among COVID-19 hospitalized adult patients in Eastern Ethiopia. Hospital-based retrospective cohort study
Source: BMC Infect Dis. 2022 Apr 28;22:412. doi: 10.1186/s12879-022-07402-6 (PMC9048613; doi:10.1186/s12879-022-07402-6)
Supplement: Supplementary file 1 — Additional file 1. Data collection tool. [file 12879_2022_7402_MOESM1_ESM.docx]

**Additional file 1: Data collection tool**

| **Part 1: Socio-demographic characteristics** | | | | | |
| --- | --- | --- | --- | --- | --- |
| **s.no** | **Questions** | **Response** | | **Remark** | |
| 1. | MRN | __________ | |  | |
| 2. | Age [years] | ___________ | |  | |
| 3 | Sex | 1. Male 2. Female | |  | |
| 4 | Region | 1. Oromia 2. Harari 3. Other | |  | |
| 5 | Ethnicity | 1. Oromo 2. Amahara 3. Harari 4. Somali 5. Other____ | |  | |
| 6 | Occupation | 1. Unemployed 2. Employed 3. Healthcare worker | |  | |
| 7 | Marital status | 1. Single 2. married 3. divorced/separated 4. widowed 5. other_____ | |  | |
| 8. | Smoking history | 1. yes 2. no | |  | |
| 9. | Alcohol drinking history | 1. Yes 2. No | |  | |
| 10 | History of medication | 1. Yes 2. No | |  | |
| **Part 2: COVID-19 symptoms, signs, laboratory finding, and image findings** | | | | | |
| 11. | Date of admission____/___/___ | | Date of death_____/______/____OR Date of discharge_______/_____/_____ | |  |
| 12. | Outcome of the patient | | 1. Recovered/discharged 2. Died | |  |
| 13. | COVID-19 symptoms on admission | | 1. dry cough 2. fever 3. SOB 4. Fatigue 5. Diarrhea 6. Asymptomatic 7. Others__________ | |  |
| 14. | COVID-19 signs on admissions | | 1. Fever______________ 2. RR_______________ 3. HR________________ 4. SPO2_______________ | |  |
| 15. | COVID-19 clinical type | | 1. Mild 2. Moderate 3. Severe 4. Critical | |  |
| 16 | Laboratory finding other than RT-PCR (recent) | | 1.LFT: AST____ ALT_______LDH____Bilirubin___  2. RFT: Cr_________BUN_______  3.Neutrophilis_______________  4.Lymphocyte___________  5. Leucocyte__________  6. Hgb_____  7. PLT____________  8. ESR__________  9. D.dimer ______________  10. Blood group______ | |  |
| 17 | Imaging finding(recent) | | 1.X-ray___________________________  2. U/S____________________________  3.ECG/Echo________________________  CT-scan____________________________ | |  |
| 18. | Comorbidities | | 1. Hypertension 2. DM 3. Heart disease 4. Renal disease (ARF,CKD..)(stage_________) 5. Lung disease 6. Cancers(any form) 7. Stroke 8. Other__________________ | |  |
| 19. | Respiratory disease | | 1. VTE 2. Pneumonia (evidenced by radiology) | |  |
| 20. . | BMI(kg/m2) | | _____________________ | |  |
| 21. | Treatment given | | 1. Did the patient receive Dexamethasone? 1. Yes, 2. No 2. Did the patient put on Mechanical ventilator? 1. Yes 2. No | |  |
| 22 | Did the patient develop organ failure | | 1. Yes 2. No | |  |
|  | **For Deceased patient** | | | |  |
| 23 | Cause of death | | **Immediate causes of death**   1. Respiratory failure(respiratory insufficiency, hypoxia, or acute respiratory distress syndrome (ARDS)) 2. organ failure 3. sepsis, septic shock 4. pulmonary embolism 5. other_____   **Underlying cause of death**_____________________________ | |  |
